# Microbiota-targeted therapeutic strategies for elderly-onset rheumatoid arthritis: based on the gut-joint axis

**DOI:** 10.3389/fimmu.2026.1850656

**Published:** 2026-05-22

**Authors:** Mengyao Liu, Weijie Wang, Leilei Qian

**Affiliations:** 1 The People’s Hospital of Rugao, Rugao, China; 2 The Second Affiliated Hospital of Zhejiang Chinese Medical University, Hangzhou, China; 3 The Department of Nephrology and Rheumatology, The People's Hospital of Rugao, Rugao, China

**Keywords:** dietary habits, elderly-onset rheumatoid arthritis, gut microbiota, gut-joint axis, immunosenescence

## Abstract

Elderly-onset rheumatoid arthritis (EORA) presents distinct clinical challenges, including more refractory disease activity, higher comorbidity burden, and increased disability and mortality compared to younger-onset RA. Emerging evidence implicates the gut-joint axis-specifically the synergistic interplay between immunosenescence, inflammaging, and gut microbiota dysbiosis-in the pathogenesis of EORA. This review aims to synthesize current evidence on the role of the gut microbiota in EORA, elucidate the mechanistic links between age-related immune changes and microbial dysbiosis, and evaluate the therapeutic potential of microbiota-targeted interventions, including dietary modifications, nutraceuticals, and fecal microbiota transplantation.

## 1 Introduction

Rheumatoid arthritis (RA) is a common autoimmune disease characterized by chronic inflammation, synovial hyperplasia, pannus formation and subsequent joint destruction, primarily affecting the peripheral joints (1). Rising global life expectancy has led to a steady increase in both the incidence of elderly-onset RA (EORA) and the prevalence of patients aging with long-standing, younger-onset RA (YORA). This demographic shift presents a growing public health challenge (2). Compared to patients with YORA, although formal EORA-specific diagnostic definitions vary across studies, those with EORA display distinct clinical characteristics, including differences in serological profiles and a higher frequency of atypical presentations. Furthermore, EORA is associated with more refractory disease activity, a greater burden of comorbidities, and increased rates of disability and mortality (3–5). This clinical divergence may be attributable to a triad of interconnected age-related conditions: immunosenescence/inflammaging, endocrine-senescence, and gut microbiota dysbiosis (6). While a triad of age-related factors has been proposed, this review will specifically focus on the synergistic interplay between immunosenescence/inflammaging and gut microbiota dysbiosis in the pathogenesis of EORA, and explore the resulting therapeutic opportunities.

Despite growing interest, the characteristics of the immune system and gut microbiota in EORA remain insufficiently explored. This review aims to summarize current knowledge on gut microbiota profiles in EORA and to elucidate the interplay between immunosenescence, gut microbiota, and aging in the context of this condition. Furthermore, we provide insights into the therapeutic potential of microbiota-based strategies for optimizing clinical management.

This review was conducted a comprehensive literature search across multiple databases, including PubMed, Web of Science, and the Cochrane Library, to identify studies published up to December 2025. A combination of Medical Subject Headings and free-text terms was used, with key search terms including: “elderly-onset rheumatoid arthritis, ” “EORA, ” “late-onset rheumatoid arthritis, ” “immunosenescence, ” “inflammaging, ” “gut microbiota, ” “gut-joint axis, ” “dietary intervention, ” “mediterranean diet, ” “fasting, ” “ketogenic diet, ” “nutraceuticals, ” “probiotics, ” “fecal microbiota transplantation, ” along with their synonyms and related variants. Non-English articles, conference abstracts without full text, and studies exclusively on younger-onset RA without relevance to aging or microbiota were excluded. After initial screening, the literature was thematically organized into three major sections including pathogenesis (immunosenescence/inflammaging and gut microbiota, mechanisms linking gut microbiota to EORA, and microbiota-targeted therapeutic strategies. This review provides a comprehensive yet focused overview of the current evidence.

## 2 Immunosenescence, inflammaging and gut microbiota in EORA

### 2.1 Immunosenescence and inflammaging

Immunosenescence refers to the progressive deterioration of both innate and adaptive immune functions with aging (7). It is characterized by thymic atrophy, T cell exhaustion and dysfunction, and alterations in the proportions and functionality of B cell subsets (8, 9). T cell senescence is a hallmark of immune aging and is driven by several factors, including the depletion of the naïve T cell pool, an imbalance in the naïve-to-memory T cell ratio, telomere erosion (which limits homeostatic proliferation), and a loss of effector plasticity (8). These alterations collectively contribute to the unique pathophysiology of RA in the elderly (10, 11). An increased frequency of senescent-like CD28^null^T cells is recognized as a hallmark of immunosenescence. The upregulation of genes associated with cellular senescence and inflammation serves as a marker for identifying senescent cells and predicting senescence-related pathways (12). Direct evidence specifically in EORA patients comes from Cezar et al. (13), who reported that compared with age-matched healthy individuals, elderly patients with RA (>60 years) exhibit a reduced number of naïve T cells and a significant accumulation of late-differentiated/senescent T cells, with the CD8^+^ T cell subset being the most susceptible to age-related changes. Additional direct evidence in older adults with RA demonstrated an increased frequency of senescent-like CD28^null^ T cells, a hallmark of immunosenescence. In addition, increased expression of NKG2D was observed in CD4^+^ T cells from the patient group. NKG2D enables T cells to kill target cells independently of antigen-presenting cells (APCs), thereby reducing self-tolerance (3, 14), which may contribute to exacerbated damage to synovial cells in RA. CD4^+^NKG2D^+^ T cells accumulate with age and are associated with enhanced immune activation and terminal differentiation (15, 16). These immunosenescence-related alterations likely contribute to the distinct pathogenic mechanisms and clinical phenotypes observed in RA patients of different age groups.

“Inflammaging” describes the complex remodeling of the immune system towards a chronic, low-grade inflammatory state, characterized by elevated systemic levels of pro-inflammatory mediators, which was originally described in general aging populations (9). Among the super-aged population, the decline in overall health is closely associated with increased levels of inflammatory markers such as C-reactive protein (CRP), interleukin-6 (IL-6), and tumour necrosis factor-alpha (TNF-α). Accardi et al. (17) demonstrate an age-related increase in the pro-inflammatory state, with the exception of semi- and supercentenarians. The regulation of inflammatory responses might represent a critical mechanism contributing to the extremely long-lived individuals. A pro-inflammatory environment may act in concert with the development of autoreactive T and B cells to promote the progression of RA. Concurrently, persistent systemic inflammation may accelerate immunosenescence and the development of additional comorbidities. In EORA, it is plausible to hypothesize that chronic low-grade inflammation serves as “fuel” that accelerates immune cell exhaustion and dysfunction, while impairing the generation and function of new, healthy immune cells. Conversely, the senescent immune system fails to respond effectively to chronic stimulation by antigen-presenting cells, potentially triggering further upregulation of pro-inflammatory cytokines, thus establishing a detrimental feedback loop (9, 18).

### 2.2 Age-related changes in gut microbiota

Evidence from animal and *in vitro* models indicates that symbiotic bacteria contribute to immune system development and epithelial cell metabolism through collaborative interactions with the host. Simultaneously, they prevent pathogen invasion by occupying ecological niches, competing for nutrients, and secreting bacteriocins. Beneficial commensal bacteria play an essential role in maintaining intestinal immune homeostasis and barrier integrity (19). Studies in animal models of aging have demonstrated that organismal aging affects intestinal epithelial remodeling, leading to a reduction in goblet cell numbers, decreased expression of tight junction proteins, and increased barrier permeability, thereby establishing a chronic, low-grade inflammatory state in the gut (20–23). Chronic inflammation accelerates intestinal aging and promotes disease progression (24, 25). Furthermore, studies in general aging populations have demonstrated that the composition of the human gut microbiota undergoes alterations with advancing age, including reduced α-diversity, diminished abundance of beneficial commensal bacteria, and expansion of potentially pathogenic and pro-inflammatory microbial populations (26). These collective changes contribute to metabolic dysregulation, accelerate immunosenescence, and disrupt intestinal barrier integrity. This renders the gut more susceptible to microbial invasion and systemic inflammaging, forming a key component of the “gut-joint axis” (27).

A comprehensive study (26) in centenarians revealed that despite geographical and lifestyle differences, they exhibit a lower abundance of beneficial commensal bacteria compared to both younger individuals and age-matched healthy peers. In contrast, the microbiota of centenarians showed an enrichment of certain health-associated genera(such as *Akkermansia* spp.), alongside an increased abundance of several opportunistic pathogens associated with disease states. This suggests a complex remodeling where beneficial taxa may be crucial for surviving the expansion of potential pathobionts. Additionally, gut microbial genes related to xenobiotic degradation were enriched in extremely long-lived populations. Centenarians represent a highly heterogeneous group: some experience healthy aging, while others live with multiple chronic diseases and frailty yet still achieve longevity. As organisms age, various microbial taxa—influenced by lifestyle, dietary habits, and other factors—guide the host along a continuum between healthy and unhealthy aging. A cohort study of long-lived individuals in Eastern China (28) analyzing gut microbiota composition indicated that longevity-associated populations are often characterized by an enrichment of specific bacterial groups, including *Clostridium* cluster XIVa, Ruminococcaceae, *Akkermansia*, and Christensenellaceae. Among these, certain taxa contribute to short-chain fatty acid production and function as beneficial bacteria that help maintain host homeostasis (29). Both *Akkermansia* and Christensenellaceae have been associated with favorable body mass index, immune modulation, and metabolic health (30, 31).

A systematic review of frail older adults evaluated differences in gut microbiota composition between frail individuals and healthy older adults (27). The findings revealed that the microbial profile of frail older adults was characterized by a reduced abundance of the phylum Firmicutes, with notable decreases in genera such as *Dialister*, *Lactobacillus*, and *Ruminococcus*. In contrast, healthy controls exhibited a higher relative abundance of Proteobacteria at the genus level. Overall, frail older adults demonstrated lower gut microbial diversity and reduced abundance of short-chain fatty acid (SCFA)-producing bacteria, potentially contributing to increased intestinal permeability and elevated pro-inflammatory cytokines. Another study (32) reported that in older individuals, duodenal microbial diversity declines with advancing chronological age, increased medication use, a higher number of comorbidities, and elevated *Escherichia coli* levels. These changes were most pronounced when comparing young adults in their 20s and 30s with adults aged 70 years and older. Therefore, for EORA, we hypothesize that age-related dysbiosis baseline and RA-specific microbial interference create a synergistic effect to form a unique pathogenesis environment.

### 2.3 Synergistic interplay

**Figure 1:**
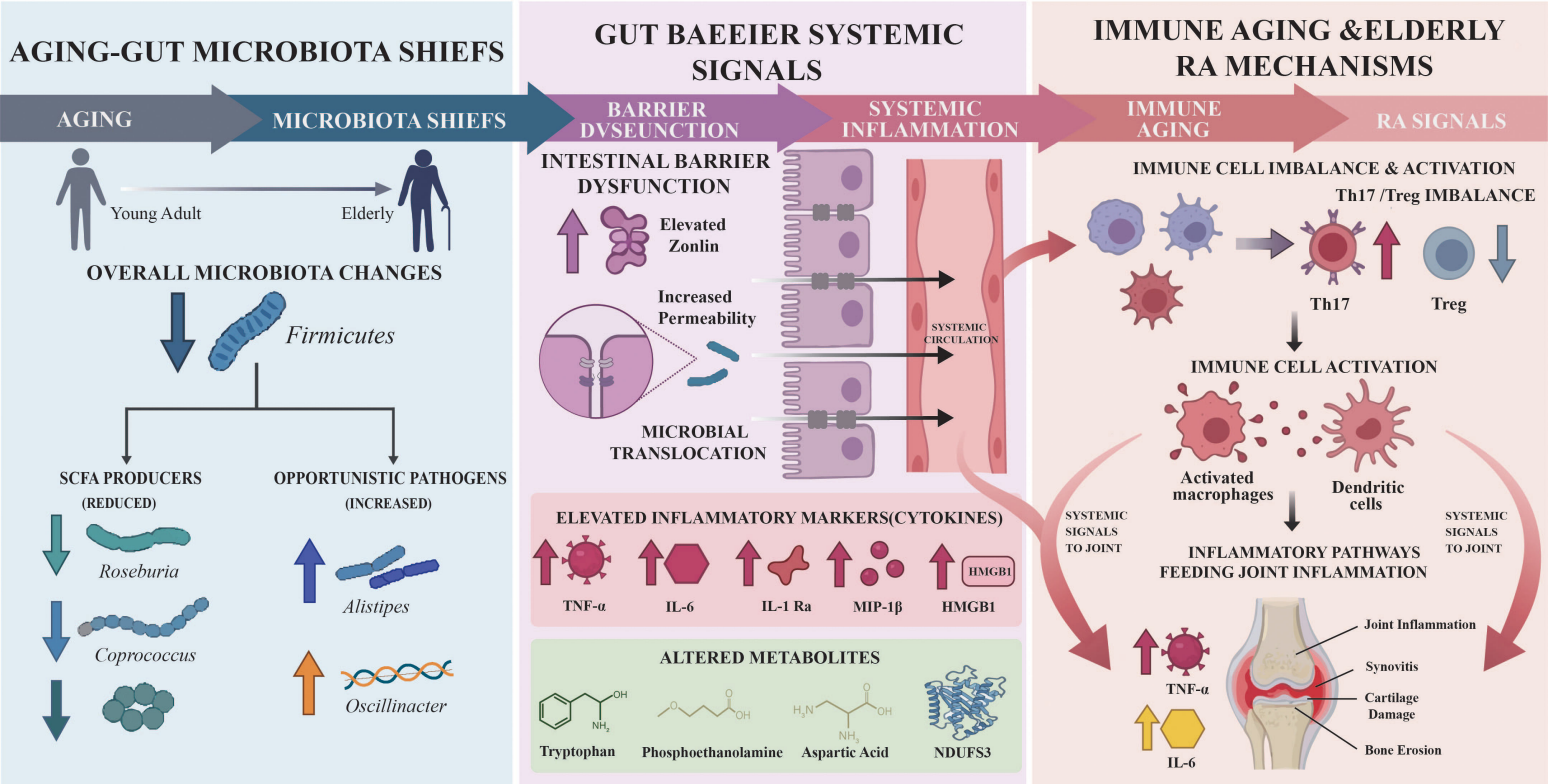
The gut-joint axis in the pathogenesis of EORA. This figure is a schematic illustration the synergistic interplay between immunosenescence, inflammaging, and gut microbiota dysbiosis. Age-related changes (e.g., thymic atrophy, intestinal barrier dysfunction) lead to a state of chronic low-grade inflammation and gut dysbiosis. This compromises intestinal barrier integrity, allowing microbial products (e.g., LPS) and potentially whole bacteria to translocate, activating local and systemic immune cells. These activated cells, including Th17 cells, migrate to the joints, where they promote synovial inflammation and osteoclastogenesis, ultimately driving the development of EORA.

Evidence from studies in other age-related diseases (obesity, type 2 diabetes, coronary heart disease) suggests that gut dysbiosis and elevated circulating lipopolysaccharide (LPS) are common features of systemic inflammation (33). This highlights a common pathway through which an aging gut can contribute to multi-morbidity. This may be one of the reasons for the increased burden of comorbidities in EORA. We propose that while gut aging and EORA share features like reduced diversity and increased pathobionts, their relationship is synergistic rather than merely parallel. Based on ex vivo studies in patients with inflammatory bowel disease (IBD) (34), age-related dysbiosis and increased intestinal permeability create a permissive environment where in a genetically susceptible individual, specific arthritogenic bacteria can flourish. This synergistically amplifies local and systemic inflammation, thereby may lowering the threshold for the development and propagation of EORA. However, extrapolated from animal models, the patterns of gut microbial imbalance differ between these two pathological states. In intestinal aging, the dysbiosis is primarily driven by host-related factors such as immunosenescence, dietary changes, and reduced intestinal motility (35). In contrast, although direct EORA evidence is lacking, in RA, the imbalance is typically induced by the enrichment of specific pathogenic bacteria that promote an increase in pro-inflammatory factors (36). Furthermore, in the aging gut of animal models, certain commensal bacteria (e.g., *Bacteroides* spp.) may increase in abundance but exhibit reduced metabolic capacity (37) (**Figure 1**).

## 3 Mechanistic links between gut microbiota and EORA

Previous studies in general patients with RA have confirmed that the gut microbiota exhibits widespread dysbiosis at the phylum level, although genus-level alterations are influenced by geography, ethnicity, age, sex, and disease duration (38–40). Based on studies in general RA populations and animal models (38, 41) found that *Prevotella* is abnormally expanded in the gut of RA patients, where it activates mucosal immune cells through pattern recognition receptors, triggering the release of pro-inflammatory cytokines such as IL-1β, IL-6, and IL-23. In animal models of arthritis, these cytokines drive the differentiation of naïve CD4^+^ T cells into pathogenic Th17 cells, which then migrate to the joints, promoting osteoclastogenesis and synovial inflammation, thereby establishing a critical link between gut dysbiosis and arthritis pathology (36, 41). While these mechanisms have not been directly studied in EORA, they are biologically reasonable. A microbiota-wide association study (MGWAS) (42) found that in untreated RA patients, certain gut microbial strains—such as *C. asparagiforme* and *Bacteroides* sp.—were positively correlated with IgA titers. In contrast, an unclassified *Lactobacillus* species was associated with serum IgG titers, potentially linking this gut bacterium to a systemic, rather than just mucosal, adaptive immune response. The same study (43) which came from a pre-RA population also indicated that the anti-CCP positive population has an overabundance of *Helicobacteraceae*, *Erysipelotrichaceae*, *Ruminococcaceae*. Additional studies have revealed a significant reduction in Firmicutes, particularly butyrate-producing bacteria (BPB), within the gut microbiome of patients with RA. Butyrate, a key SCFA, possesses a wide range of physiological functions. An imbalance between butyrate-producing and butyrate-consuming bacteria has been observed in the gut microbiome of RA patients, a phenomenon associated with levels of anti-citrullinated protein antibodies (ACPA) and joint deformity, however, it was a cross-sectional association, causality not established. Butyrate may exert its effects by inhibiting histone deacetylase (HDAC) expression and the transcription of pro-inflammatory cytokine genes, thereby promoting Tregs while simultaneously suppressing conventional T cells and osteoclast activity (44, 45). Preclinical evidence from collagen-induced arthritis(CIA) models has demonstrated that dietary butyrate possesses anti-inflammatory properties and modulates T cell balance.

## 4 Microbiota-targeted therapeutic strategies for EORA

**Figure 2:**
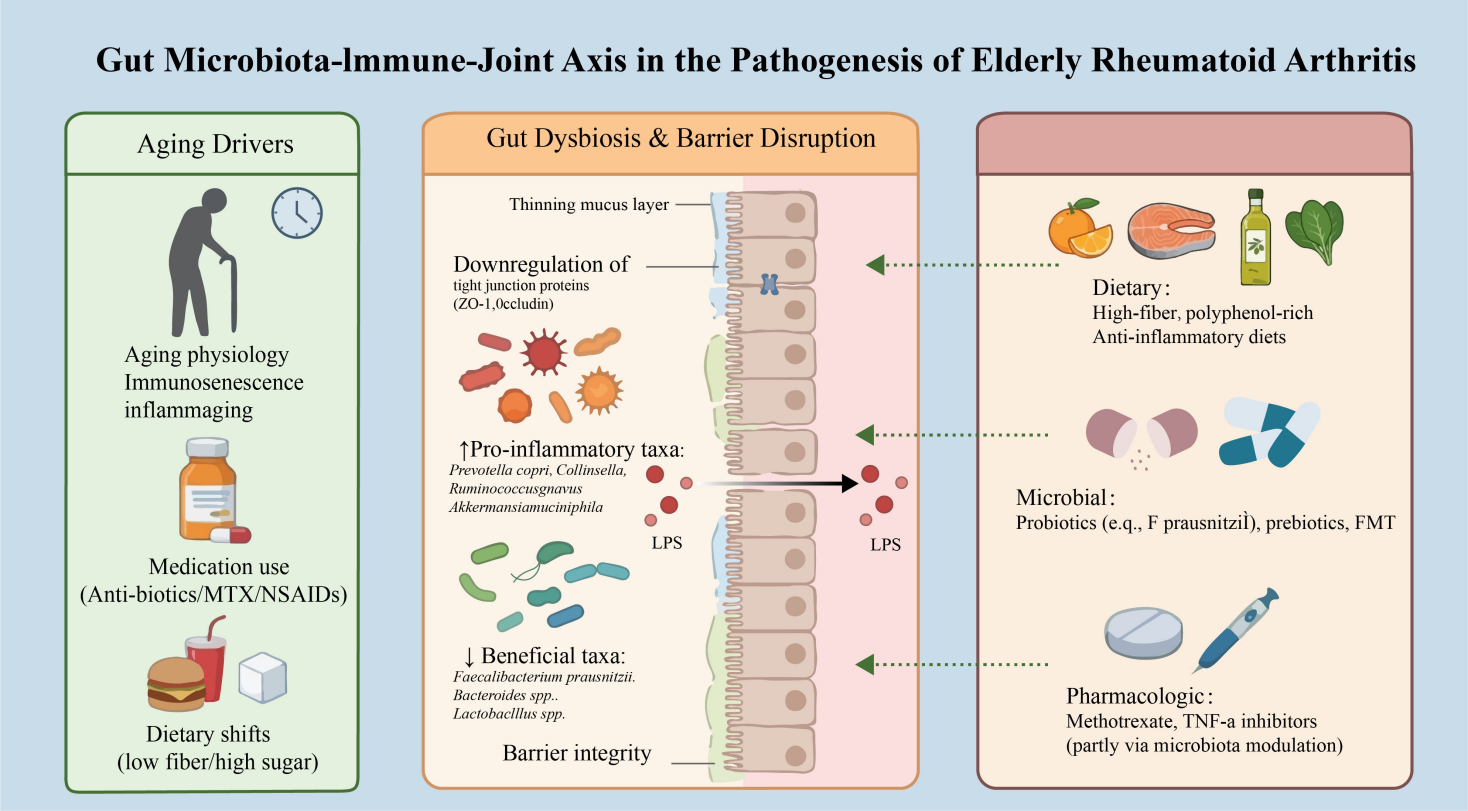
Microbiota-targeted therapeutic strategies for EORA. This schematic overview categorizes therapeutic strategies by their site and mechanism of action. Dietary patterns, specific nutritional components, nutraceuticals and live biotherapeutic can act on the gut ecosystem. These interventions aim to increase beneficial bacteria and SCFA production, decrease pathobionts and pro-inflammatory mediators, and restore intestinal barrier integrity. These changes can reduce systemic inflammation and improve disease activity in EORA.

The clinical management of EORA is complicated by the perceived risk of adverse events from conventional and biologic DMARDs in older adults. More recent studies have confirmed that this difference of treatment persists in the era of biologic and targeted synthetic DMARDs, although the magnitude may have diminished (46). Studies show that even with comparable disease activity and comorbidity profiles, physicians often prescribe less intensive regimens for older patients compared to younger ones (47) (**Figure 2**). This “treatment gap” underscores the urgent need for effective and well-tolerated adjunctive or alternative strategies, such as those targeting the gut microbiota (**Table 1**).

**Table 1:** Summary of main effects and evidence strength for microbiota-targeted interventions in EORA.

Intervention category	Specific intervention	Main Effects	Study Design	Evidence Source Type	Ref.
Whole Dietary Patterns	MD	Improvements in morning stiffness, number of swollen joints, pain scores and physical function; Reduce CVD risk; Facilitat higher response rates to antirheumatic drugs Reduce *Lactobacillaceae* and *Prevotella*	RCT; observational studies	Extrapolated from general RA (with some age subgroup analyses)	(48–50, 52–57, 59–62)
	IF	Reduce DAS-28 and MDA Weight loss	RCT in overweight/obese postmenopausal women with RA (age 50–70 years)	Extrapolated from general RA (age 50–70 years, partially overlaps with EORA)	(64–66, 69–72)
	KD	Weight loss Metabolic changes	Small studies in general RA (non-elderly); animal models	Extrapolated from general RA (non-elderly) +animal models	(67, 73–77)
	Vegan diets	Modest pain improvement	RCT; observational studies	Extrapolated from general RA	(78–83)
Specific Foods, Nutrients	Fish/omega-3 PUFA	Increased numbers of small VLDL particles Metabolic changes Reduce RA risk Improve disease activity	Meta-analyses of cohort and case-control studies	Extrapolated from general RA (older adults included)	(84, 85)
	Red/processed meat restriction	Increase RA risk with high intake; Stronge association of greater risk of disease activity in overweight/smokers	Case-control and cohort studies	Extrapolated from general RA (some studies include older women)	(86–89)
	Flaxseed	Reduce DAS-28 Prolonge bleeding time	RCTs with conflicting results	Extrapolated from general RA	(90, 91)
	Curcumin	Reduce the number of swollen, tender joints, ESR, hs-CRP, VAS, DAS-28, insulin resistance (HOMA-IR), triglyceride levels, and BMI; Improve TAC	RCTs	Extrapolated from general RA (age 25–70 years)	(92, 93)
	Flavonoids	Reduce RA risk, particularly prominent among individuals aged≥60 years, non-drinkers, and those without a history of cardiovascular disease	Epidemiological studies; general US population (≥60 years subgroup shows stronger protection)	Extrapolated from general population (≥60 years subgroup analysis available)	(94, 95)
	Cinnamon	Reduce joint tenderness and swelling, TNF-α, and CRP	RCT	Extrapolated from general RA (mean age 50 years)	(96)
Beverages	Soy milk / isoflavones	Reduce TNF-α, hs-CRP;	RCT; a cross-sectional study; animal models	Extrapolated from general RA + animal models	(97, 98)
	Coffee	Regulate the Th1/Th2 cell balance Increase *Bifidobacterium, Faecalibacterium*	Observational studies	Extrapolated from general RA + healthy volunteers	(99–103)
	Cranberry juice	Reduce ESR, CRP, DAS-28, adiponectin, IL-6 (Combined with fish oil)	RCT	Extrapolated from general RA (mean age 55 years)	(104)
	Sugar-sweetened beverages	Improve risk of seropositive RA, especially late-onset (≥55 years)	Prospective cohort	Extrapolated from general female population (late-onset ≥55 year)	(105)
Micronutrients	Vitamin E	Nonlinear inverse association with all-cause mortality (intake <7.097 mg/day)	NHANES cohort	Extrapolated from general RA (NHANES, age ≥20 years, RA subgroup)	(106)
	Zinc	Reduce incidence of osteopenia or osteoporosis in RA Improve cIMT in some subgroups	Cross-sectional and cohort studie	Extrapolated from general RA (age >60 years subgroup in one study)	(107, 108)
	Magnesium	Inverse association with all-cause mortality; strongest in women with RA, age <65 years, BMI ≤30 kg/m²	NHANES cohort	Extrapolated from general RA	(109)
Nutraceuticals	Synbiotics	Reduce DAS-28, hs-CRP, VAS, oxidative stress markers(NO and GSH), insulin levels and HOMA-IR; Improve in total cholesterol, LDL-C, and the LDL/HDL ratio	RCT	Extrapolated from general RA mean age 49 years)	(110)
	Probiotics alone	Some results show reduce TNF-α, IL-6, CRP and increase IL-10, others no benefit	RCTs; meta-analysis	Extrapolated from general RA	(111–113)
	CoQ10	Reduce MDA, TNF-α, ESR, DAS-28	RCTs and systematic review	Extrapolated from general RA	(114–116)
FMT		Reduce DAS-28 in single case	Case report (young RA); animal studies	Animal models + single case report (young RA)	(117–119)

MD: Mediterranean diet; DAS-28: Disease Activity Score in 28 joints; CRP: C-reactive protein; CVD: Cardiovascular disease; RA: Rheumatoid arthritis; EORA: Elderly-onset rheumatoid arthritis; IF: intermittent fasting; MDA: Malondialdehyde KD: Ketogenic diet; VLDL: Very Low-Density Lipoprotein; VAS: Visual Analog Scale; TAC: Total antioxidant capacity; TNF-α: Tumour necrosis factor-alpha; NHANES: National Health and Nutrition Examination Survey; cIMT: Carotid intima-media thickness; HOMA-IR: Homeostatic Model Assessment for Insulin Resistance; IL-6: interleukin-6; ESR: Erythrocyte sedimentation rate; LDL-C: low-density lipoprotein cholesterol; CoQ10: Coenzyme Q10; FMT: Fecal microbiota transplantation. References.

### 4.1 Whole dietary patterns

#### 4.1.1 Mediterranean diet

The Mediterranean diet (MD), a dietary pattern based on plant-derived foods with moderate consumption of fish and wine, while limiting processed meats and sugary products, was recommended as a preventive strategy in the 2022 RA guidelines (48). Compared to other dietary patterns, MD demonstrated higher frequency and intake of antioxidant-rich foods, various vitamins, and n-3 fatty acids (49, 50). Alawadhi et al. found (51) that RA patients with Disease Activity Score in 28 joints (DAS-28) < 3.2 predominantly consumed olive oil, vegetables, fruits, and legumes. In contrast, patients in the cohort with DAS-28 ≥ 3.2 consumed more red meat, butter, sugar-sweetened beverages, and refined pastries. Evidence from general RA populations has shown that sustained MD adherence was associated with significant improvements in morning stiffness, number of swollen joints, and pain scores (52, 53), and is associated with lower disease activity, better physical function, and improved quality of life with beneficial effects even observed in individuals with RA-associated cachexia (54–57). In a prospective multicentre cohort study of 193 patients with chronic inflammatory diseases initiating biologic therapy, Overgaard et al. found that in the RA subgroup, patients adhering to such a diet were nearly ten times more likely to achieve clinical response. However, due to the very small sample size, it cannot be used as a definitive basis, which warrants further investigation in randomised controlled trials (58). Notably, based on Overgaard’s research, it can be speculated that differential responses to dietary fiber and meat intake may indicate that RA is an autoimmune disease more susceptible to dietary factors, which may have important implications for the development of dietary-assisted therapies for RA and even EORA. In addtion, due to its anti-inflammatory properties, MD is associated with reduced cardiovascular disease (CVD) risk, and the interaction between CVD and MD may contribute to decreased RA incidence, rendering the MD particularly suitable for elderly RA patients with concurrent cardiovascular conditions (59, 60). Sex differences exist in the MD’s effects: Hu et al. (60) found no significant association with RA risk in women, while Johansson et al. (61) observed an inverse correlation between MD scores and RA risk in seropositive men, further analysis suggested that this association was partially mediated by smoking status. Nguyen et al. (62) following 60, 000 women, reported that adherence to the MD reduced RA risk in women who were regular smokers. From a microbiota perspective, patients with high adherence to the MD exhibit reduced *Lactobacillaceae* and nearly undetectable levels of *Prevotella *(63). For individuals with elderly RA, whose gut microbial diversity diminishes under the influence of both aging and disease, adopting an MD may help ameliorate RA through modulation of the intestinal microbiota. It is important to note, however, that most MD studies have been conducted in general RA populations (mean age 50–65 years). Direct evidence specifically in EORA patients remains limited, although the safety and cardiovascular benefits of MD support its use in older adults.

#### 4.1.2 Intermittent fasting, ketogenic diet and vegan diet

While the MD represents a sustainable, long-term dietary pattern, intermittent and modified fasting regimens have gained attention for their potential to induce rapid, albeit transient, shifts in immune and metabolic parameters. Intermittent fasting (IF) encompasses a variety of different protocols that vary in fasting length and fluid intake. Methods such as time-restricted eating, alternate fasting, and hydration are associated with weight reduction and metabolic benefits. A RCT involving overweight and obese postmenopausal women with RA aged 50–70 years showed that intermittent fasting significantly reduced body weight, along with marked decreases in DAS-28, CDAI, and Health Assessment Questionnaire (HAQ) scores (64). Two additional RCTs focusing on similar populations corroborated these findings and further indicated that this dietary pattern helps reduce malondialdehyde (MDA, a lipid peroxidation marker), inflammatory parameters, and liver enzyme level (65, 66). This population partially overlaps with EORA age range, but formal EORA criteria were not applied. The underlying mechanisms may involve decreased serum IL-6, modulation of CD4^+^ T-cell function, or improved IgG glycosylation status (67, 68). These studies provide some of the strongest direct evidence for dietary interventions in older RA patients, although evidence for patients aged >70 years remains extrapolated. A unique form of IF is fasting during Ramadan, which involves approximately 15.5–16.5 hours of daily fasting, not even drinking water. Several studies have demonstrated significant reductions in DAS-28 scores and inflammatory levels post this type of IF, with beneficial effects persisting for up to three months, although none of these studies specifically enrolled EORA patients, the mean ages ranged from approximately 45 to 55 years (69–71). However, given the age-specific risks, especially in patients with blunted thirst perception or concomitant use of diuretics, or in patients with diabetes who are at risk of concomitant use of hypoglycemic drugs, medication application can obscure early warning symptoms. In addition, imbalances in nutrient intake during a short eating window may exacerbate sarcopenia and weakness. Therefore, the application of this model needs to be considered with caution. Despite these considerations, the available evidence supports the anti-inflammatory potential of fasting during Ramadan in patients who choose to adhere to this religious practice.

Compared to strict fasting regimens, ketogenic diets (KD) are often reported to have higher rates of patient acceptance and adherence, although research on KD in RA remains limited. While some studies have observed that KD induces an acute decrease in insulin-like growth factor-1 (IGF-1), no association has been found with reductions in T-cell numbers or function (73). Similar to fasting regimens, KD induces metabolic and hormonal changes, with its anti-inflammatory properties potentially mediated primarily through β-hydroxybutyrate (BHB) production. BHB may exert anti-inflammatory effects through multiple mechanisms: inhibiting NLRP3 inflammasome-mediated IL-1β and IL-18 release (74), reducing TNF-α expression, suppressing IL-1 synthesis in macrophages and neutrophils (75, 76) and, via HCAR receptor activation, inhibiting the production of pro-inflammatory cytokines including IL-1, IL-12, and IL-6 (77). KD is fundamentally characterized by low carbohydrate intake. A systematic review and meta-analysis of clinical trials demonstrated that low-carbohydrate diets significantly reduce body weight, body mass index (BMI), waist circumference, blood pressure, plasma triglycerides, fasting glucose, glycated hemoglobin, plasma insulin, and C-reactive protein levels, while increasing high-density lipoprotein cholesterol (HDL-C) (120). These findings suggest potential benefits for cardiovascular risk factors. However, some studies suggest that KD’s cardiovascular protective effects may be minimal. Currently, evidence regarding KD’s impact on cardiovascular health in patients with inflammatory arthritis remains lacking. Nevertheless, its weight reduction benefits may offer clinical value in RA management. Direct evidence for KD in RA is limited to small or non-elderly cohorts. Due to the lack of studies on EORA, perhaps this model can be tried with caution for ultra-heavy EORA with combined cardiovascular risk.

Both the MD and fasting protocols often encourage a higher intake of plant-based foods. This raises a distinct question: can the complete exclusion of animal products, as seen in vegetarian and vegan diets, offer additional or unique benefits for EORA? Early randomized controlled trials involving 34 RA patients who followed a fasting-to-vegetarian dietary pattern demonstrated no adverse effects on overall nutritional status (78). However, based on these findings, the research team observed that when patients’ dietary patterns changed, significant alterations in the gut microbiota also occurred (79). Compared to high-meat diets, transitioning from a mixed diet to a vegan diet resulted in decreased numbers of neutrophils, monocytes, and platelets. Consistent with previous research, when vegetarians are already at risk of micronutrient deficiencies, this may lead to declines in immune parameters (80). Menzel et al. (81) conducted a cross-sectional study including 36 vegetarians and 36 omnivores; although they could not confirm a precise relationship between vegetarianism and inflammatory factors, they found that the duration of strict vegan diet was positively correlated with resistin, IL-18, and IL-1RA—key catalysts in inflammatory processes (121, 122). They also observed that long-term vegans (>4.8 years) were more likely to have lower hs-CRP levels, consistent with findings from Haghighatdoost (82). A recent meta-analysis encompassing 7 RCTs spanning 40 years (83) examining the effects of vegetarian diets on RA patients found that vegetarian diets only yielded modest improvements in pain intensity, with insufficient evidence to provide clinical recommendations regarding disease activity or physical function. Given the conflicting conclusions across different studies, further research is needed to explore the relationship between duration of vegetarian diets and inflammatory status. Meanwhile, since the included RA population involved all age and no subgroup of elderly studies were conducted, further validation is needed to determine whether the conclusions are also applicable to EORA.

Although the potential benefits of dietary modification for directly reducing inflammatory markers and disease activity in patients with RA remain controversial, a growing body of evidence suggests that it confers sustained positive impacts through indirect pathways. These benefits are primarily mediated by improvements in glycemic and lipid profiles, reductions in body weight, and enhanced physical activity levels, which collectively contribute to ameliorating RA disease activity. Among all lifestyle factors associated with extreme longevity and health, dietary patterns—particularly a high-fiber diet—are among the most frequently discussed (123). Maintaining or promoting microbial diversity through dietary or other interventions may be a worthwhile strategy to explore in the context of elderly RA. However, clinical trials investigating dietary interventions are challenging to design and implement, and their results are often heterogeneous due to variations in intervention types, duration, and patient compliance. Following dietary interventions, patients with RA appear to undergo a subtle yet lasting shift in their subsequent dietary patterns, often adopting healthier food choices in the long term (124). To move towards more precise dietary recommendations, it is essential to dissect these patterns and understand the specific effects of their constituent parts.

### 4.2 Specific foods, nutrients

Evidence from general RA populations has identified high consumption of processed or red meat as an independent risk factor for RA development, an association that appears consistent across age, sex, and ethnicity (86). For individuals who are overweight or smokers, high intake of processed or red meat is associated with an even greater risk of disease activity in smoking-adjusted analyses (86). Women with RA tend to be more cautious about red meat consumption, possibly due to concerns regarding RA risk (125, 126). This caution appears justified: a study focusing on older women demonstrated that following red meat consumption, women with RA exhibited significantly higher phenylalanine concentrations in both fasting and postprandial samples (87). Bäcklund et al. (88)conducted a nested case-control study quantifying the intake of key dietary components and the risk of developing RA, reporting a strong association between increased consumption of red or processed meat and the risk of seropositive RA. The cooking of red meat may enhance oxidative stress and inflammatory processes (127). Its iron content has been linked to upregulation of pro-inflammatory mediators such as IL-6, IL-8, IL-1β, and TNF-α (128), while other research suggests that arachidonic acid in red meat may trigger the production of inflammatory factors (129). Furthermore, gut microbiota-dependent catabolism of red meat may promote inflammatory diseases (86). However, a case-control study by Hatami et al. involving 100 newly diagnosed RA patients and 197 healthy controls found that only higher consumption of processed meats—not red meat—was associated with increased RA risk (89). Randomized controlled crossover trials focusing predominantly on middle-aged and elderly RA populations also failed to find a correlation between red meat and RA (130, 131). Instead, they observed that fatty fish, compared to other protein sources, was positively associated with increased numbers of small Very Low-Density Lipoprotein(VLDL) particles, exhibited faster metabolism, and led to greater elevations in beneficial metabolites. Therefore, it is speculated that, for EORA, fatty fish intake may not only reduces cardiovascular disease risk but also offers greater protein gains from rapid digestion (132). Fish exerts anti-inflammatory properties through omega-3 polyunsaturated fatty acids (PUFAs). A prospective cohort study utilizing the Swedish Mammography Cohort (1987 and 1997) involving 32, 000 women found that consuming 1–3 servings of fish per week reduced RA risk (84). A meta-analysis of 7 cohort and 6 case-control studies (85)indicated that each 100 g/day increase in fish consumption reduced the risk of developing RA in middle-aged and older adults by 15%. The lowest risk was observed at an intake of 20–30 g/day. A prospective study involving 208 RA patients (133) identified high monounsaturated fatty acid (MUFA) intake as an independent predictor of low disease activity in the RA group. Given the characteristics of the study population, increasing daily MUFA intake appears particularly beneficial for older women with RA. Shellfish, which similarly increase omega-3 fatty acid intake, have also been shown to improve disease activity in women with RA (134, 135).

### 4.3 Bioactive phytochemicals

This invites investigation into whether plant-based sources of similar compounds, or other bioactive plant molecules, can exert comparable anti-inflammatory effects. Flaxseed, as a rich source of long-chain omega-3 fatty acid precursors, may also exert protective effects on RA symptoms. Ghaseminasab-Parizi et al. (90) demonstrated that flaxseed consumption for 12 weeks reduced disease activity in RA patients, as measured by DAS28-ESR(erythrocyte sedimentation rate), pain severity, and quality of life. However, a similar study failed to replicate these findings and additionally identified a potential risk of prolonged bleeding time. Consequently, flaxseed supplementation is not recommended for RA patients with bleeding risk but may serve as an adjunctive therapy for those with concomitant cardiovascular disease.

The variability in response to flaxseed highlights a common challenge in nutritional research: the complexity of whole-food interventions can obscure the effects of individual compounds. In contrast, isolated phytochemicals that directly target inflammatory signaling pathways may offer more predictable and controllable anti-inflammatory effects. Curcumin, a polyphenolic antioxidant, has demonstrated favorable effects in improving inflammatory markers in RA. Daily supplementation with 500 mg of curcumin for eight weeks significantly increased serum total antioxidant capacity (TAC) while reducing swollen/tender joint counts, Visual Analog Scale (VAS) pain scores, and DAS28 scores in female RA patients, though the mean age of population was not specified, studies included postmenopausal women (92, 93). Notably, curcumin was well-tolerated even at high doses (1200–2100 mg daily) in these RA studies, suggesting a favorable safety profile for long-term use in elderly patients, although direct EORA-specific trials are lacking. However, caution is warranted, as studies in other populations (e.g., patients with advanced colorectal cancer) have reported adverse effects like nausea and diarrhea at similar doses, indicating that safety may be context-dependent and requires further investigation (136). The success of curcumin, as a representative of the curcuminoid subclass within the polyphenol family, has sparked widespread interest in another, even larger and more ubiquitous polyphenol subclass-–flavonoids. Flavonoids, widely present in various plant-based foods such as green tea, berries, citrus fruits, and bulb vegetables, have been the subject of epidemiological investigations (94). In contrast, direct evidence for flavonoids in older adults comes from a US population-based study of Chen et al., which provided direct epidemiological evidence supporting the potential benefit of flavonoid-rich diets specifically in older adults at risk for RA (95).

The anti-inflammatory effects exhibited by flavonoids through daily dietary intake suggest that we should turn our attention to sources of phytochemicals that exist in a more concentrated form. Culinary spices are precisely such a source—they provide a high concentration of bioactive molecules in smaller intake volumes. Cinnamon is one such example, its anti-inflammatory potential having been confirmed in general RA populations. Shishehbor et al. (96) first demonstrated in female RA patients that daily supplementation with 2000 mg of cinnamon for eight weeks significantly reduced joint tenderness and swelling through inhibition of TNF-α or CRP. The anti-inflammatory effects of cinnamon’s active components, such as cinnamaldehyde and polyphenols, have been confirmed in various animal models.

### 4.4 Beverages

Our dietary intake, however, is not limited to solid food. The beverages we consume represent a significant and often overlooked source of both potentially beneficial and harmful bioactive compounds. Soy and its derivatives have shown anti-inflammatory potential. A small RCT in which approximately half of the participants were postmenopausal women reported that sustained consumption of soy milk for eight weeks was associated with significant reductions in TNF-α and hs-CRP levels compared to dairy milk control (97). While these findings suggest potential anti-inflammatory effects of soy milk in RA, the small sample size and lack of EORA-specific analysis warrant cautious interpretation. Meanwhile, although this study used dairy products as a control and found no improvement in inflammatory status, a cross-sectional study observed that daily consumption of at least one serving of dairy products was inversely associated with the prevalence of EORA in the US population (137). Soy isoflavones, as the primary active components of soybeans, have demonstrated potent anti-inflammatory effects. In RA animal models, soy isoflavones exhibited anti-arthritic, antioxidant, and lipid-lowering properties while maintaining antioxidant system homeostasis (98). These findings provide valuable references for dietary choices in elderly patients with RA. However, a meta-analysis by Asoudeh et al. (85) found no significant association between dairy product consumption and RA development in European, Asian, and US populations (particularly among women), suggesting that population heterogeneity may influence intervention outcomes.

In RA patient populations with a mean age of approximately 55 years, coffee consumption has not shown a significant association with RA risk (99). However, it may exert anti-inflammatory effects through modulation of the gut microbiota, with potential benefits for maintaining intestinal health in elderly patients. Healthy individuals consuming three cups of coffee daily for three weeks showed increased abundance of Bifidobacterium in the gut microbiota (100). The diverse bioactive compounds in coffee synergistically scavenge free radicals, inhibit NF-κB, and activate Nrf2 (138), Sirtuin-1 and PPAR-γ (139), thereby suppressing pro-inflammatory cytokines and matrix metalloproteinases while regulating the Th1/Th2 cell balance (101). Additionally, coffee may reshape gut microbial composition and diversity: habitual coffee drinkers exhibit higher abundance of Bacteroides, Porphyromonas, and Prevotella (102), and caffeine intake is associated with increased α-diversity of anti-inflammatory genera such as *Faecalibacterium* and *Alistipes* in the colonic mucosa (140). Importantly, previous research has confirmed that caffeine consumption does not affect drug efficacy or exacerbate disease activity in RA patients receiving methotrexate (MTX) therapy (103). This is clinically relevant for EORA patients who may be concerned about potential interactions between dietary caffeine and their antirheumatic medications. The effects of coffee on intestinal barrier function, while suggested by mechanistic studies (100, 102, 140), require direct investigation in EORA.

Cranberry juice, rich in polyphenolic compounds, possesses antioxidant and anti-inflammatory properties that help regulate systemic inflammatory status and may indirectly modulate immune responses through effects on the intestinal microenvironment. A randomized study demonstrated that concurrent consumption of fish oil and cranberry juice further reduced ESR, CRP, DAS28-CRP, adiponectin, and IL-6 levels (104), suggesting enhanced anti-inflammatory effects of fish oil. As a natural food source, cranberry juice is well-accepted with favorable safety profiles, making it particularly suitable for moderate dietary incorporation in elderly patients as part of comprehensive anti-inflammatory management. Furthermore, high-sugar diets may represent a potential risk factor for RA onset in elderly women. A prospective follow-up study by Hu et al. (105) involving 3, 381, 267 women found that daily consumption of ≥1 serving of sugar-sweetened carbonated beverages was associated with increased risk of seropositive RA in women, with a particularly strong correlation in late-onset RA (≥55 years). These findings underscore the importance of reducing high-sugar intake in elderly populations.

### 4.5 Micronutrients

Beyond these complex bioactive molecules, the diet provides essential micronutrients that serve as cofactors for critical enzymatic pathways. Their status in the elderly can significantly influence inflammatory and metabolic processes. In elderly RA patients, certain micronutrients exhibit pronounced protective effects. Nordström et al. (91) postulated that the discrepant effect of flaxseed supplementation might be attributable to low serum zinc concentrations among participants, as zinc is an essential nutrient for desaturase enzymes involved in the initial step of prostaglandin synthesis. Direct evidence in older RA patients comes from Fang et al. (107) who reported that higher dietary zinc intake in RA patients over 60 years was associated with reduced incidence of osteopenia or osteoporosis. However, a cross-sectional study by Vera et al. (108) identified zinc as a predictor of increased carotid intima-media thickness (cIMT) in RA patients. Notably, a significant negative correlation between zinc and cIMT was observed specifically in male patients with concomitant metabolic disease (MetD). Given the differences in inflammatory status between these conditions, whether the relationship between zinc and cIMT is influenced by the distinct inflammatory profiles of RA and MetD warrants further investigation. Furthermore, a nonlinear protective relationship exists between dietary vitamin E intake and all-cause mortality in RA patients. Analysis based on data from 2, 906 RA patients aged ≥20 years who participated in the National Health and Nutrition Examination Survey (NHANES, 1999–2018) revealed that when daily vitamin E intake was below 7.097 mg, all-cause mortality decreased with increasing intake (106). This finding suggests that moderate vitamin E supplementation may positively influence long-term prognosis in elderly RA patients, with mortality showing an inverse correlation with intake below this threshold. Similarly, adequate dietary magnesium intake is associated with reduced all-cause mortality risk in RA patients. This benefit is particularly pronounced in specific subgroups: women, individuals aged <65 years, and those with BMI ≤30 kg/m² demonstrate the most significant protective effects (109).

### 4.6 Nutraceuticals

While modifying whole diets and their components is the most natural approach, achieving therapeutic concentrations of specific bioactive compounds can be challenging through diet alone. This has led to considerable interest in the use of concentrated nutraceuticals and supplements. Gut microbiota modulators, including probiotics, prebiotics, and their combinations (synbiotics), have demonstrated potential value in regulating disease activity in RA. In an 8-week randomized double-blind placebo-controlled trial involving 54 patients with active RA, Zamani et al. (110) found that patients exhibited significant improvements in hs-CRP, DAS-28, VAS pain scores, and oxidative stress markers, along with reductions in insulin levels and HOMA-IR. Although promising, the study’s mean participant age was approximately 49 years, and no EORA-specific analysis was performed. Therefore, extrapolation of these findings to EORA requires caution and dedicated investigation. Additionally, Schaafsma et al. (141) observed that healthy volunteers receiving a combination of prebiotics and probiotics for three weeks showed significant decreases in total cholesterol, low-density lipoprotein cholesterol (LDL-C), and the LDL/HDL ratio. However, the efficacy of probiotics administered alone remains controversial. Some studies have reported that probiotic supplementation in female RA patients increases levels of the anti-inflammatory cytokine IL-10 while reducing pro-inflammatory factors such as TNF-α and IL-6, thereby improving inflammatory status (111). A meta-analysis encompassing 10 RCTs with a total of 632 RA patients also demonstrated that probiotic interventions reduced CRP levels (112). Conversely, not all studies have observed clear clinical benefits from probiotic supplementation alone in RA patients (142). These effects were influenced by confounding factors such as age, disease duration, and medication regimens. Synbiotics may exert anti-inflammatory effects through metabolic and oxidative stress pathways, but study outcomes exhibit considerable heterogeneity due to variations in intervention duration, strain selection, and dosage regimens.

Coenzyme Q10 (CoQ10), a potent antioxidant, has demonstrated potential for reducing oxidative stress and inflammation in general RA populations. Studies involving RA patients revealed that CoQ10 supplementation significantly reduced malondialdehyde (MDA), a marker of oxidative stress, suppressed TNF-α overexpression, and was accompanied by decreased serum matrix metalloproteinase activity (114, 115). A synthesis of 15 studies (116)confirming that CoQ10 supplementation reduced disease activity indices, ESR, cytokine levels, and methylglyoxal in RA patients. In animal models of arthritis, long-term CoQ10 supplementation combined with omega-3 polyunsaturated fatty acids facilitated the restoration of cellular energy metabolism and antioxidant status (143). Although direct evidence specifically in EORA patients is currently lacking, the favorable safety profile of CoQ10 suggests it may be suitable for older adults. And recent advances in nanoparticle delivery systems have enhanced the targeting and anti-inflammatory efficacy of CoQ10, significantly reducing pro-inflammatory cytokine levels and markers of macrophage activation, perhaps offering novel therapeutic approaches for elderly RA patients (144). In contrast, other antioxidants such as alpha-lipoic acid (ALA) have failed to demonstrate significant RA-ameliorating effects in clinical studies. This discrepancy highlights that antioxidant interventions are not uniformly effective and may require targeting specific inflammatory pathways or patient subgroups. Collectively, these findings indicate that CoQ10 holds promise for improving inflammatory status and disease activity in elderly RA patients; however, its efficacy may be influenced by factors including intervention duration, formulation, and concomitant medication use.

### 4.7 Fecal microbiota transplantation

Prebiotics, probiotics, and even potent antioxidants like CoQ10 work with the existing microbial ecosystem. A more radical approach is to replace the entire ecosystem itself, a concept that represents the extreme end of microbiota-targeted therapy. Fecal microbiota transplantation(FMT) is a therapeutic strategy for reconstituting the intestinal microecology. However, evidence for FMT in RA is currently limited to preclinical studies and a single case report. In animal models, FMT may improve immune balance by restoring intestinal microecology, promoting regulatory T cell (Treg) proliferation, and reducing the Th1/Th2 ratio (117). Furthermore, a large-scale animal study demonstrated that long-term preservation of “young microbiota” significantly improved peripheral and central immune status while reversing age-associated cognitive decline (118), suggesting that “young microbiota transplantation” may represent a novel strategy for delaying RA progression. A case report from China (119) provided the first evidence that FMT, through reconstruction of beneficial gut microbiota, markedly improved disease activity and reduced medication dependence in a young RA patient with poor response to multiple drugs, offering preliminary clinical support for FMT application in RA treatment.

It is important to emphasize that direct evidence specifically in EORA patients is completely lacking. While FMT may theoretically benefit elderly RA patients, particularly those with intestinal dysfunction, polypharmacy, or inadequate responses to conventional therapies, but this point remains highly speculative. Clinical studies specifically targeting elderly populations are urgently needed, and critical issues including safety profiles, long-term efficacy, and donor selection also require urgent investigation.

## 5 Discussion

This review explores the possible role of gut microbiota and immune-inflammatory interactive effect in the pathogenesis of EORA, and cautiously evaluating the potential of microbial-targeted therapeutic strategies. Immunosenescence in this process characterized by CD28^null^ T cell accumulation and CD4^+^NKG2D^+^ T cell expansion, which may distinguish EORA from healthy aging and YORA (13). Second, gut microbiota diversity is reduced in older adults(reduced α diversity, decreased SCFA-producing bacteria), as demonstrated in frail older populations (27), and similar patterns are observed in extrapolated RA studies, particularly *Prevotella* expansion (38, 41). The gut-joint axis, involving gut-derived Th17 cell migration and osteoclastogenesis, provides a mechanistic link. Third, among microbiota-targeted interventions, MD and IF have shown positive significance in targeted microbial therapy, while nutritional products and FMT need to be further validated in the specific population of EORA.

When comparing the clinical effects of different intervention types, it was found that MD was supported by several randomized controlled trials demonstrating improvements in disease activity, inflammatory markers, and cardiovascular risk factors, with the added advantage of extensive safety data in older adults. IF showed impressive results in overweight/obese postmenopausal RA women, including benefits lasting up to three months (64–66). Synbiotic supplementation improves hs-CRP, DAS28, and oxidative stress markers in elderly RA patients (110). And higher intakes of zinc, vitamin E, and magnesium are associated with reduced osteoporosis risk and lower all-cause mortality in older RA patients (106, 107, 109). In contrast, nutraceuticals such as curcumin, CoQ10, and probiotics have rarely been analyzed in age-stratified subgroups, and there was significant heterogeneity between studies due to differences in dosage, type, duration of treatment, and concomitant medications. Notably, conflicting findings exist for flaxseed and probiotics. Similarly, some probiotic RCTs have shown reduced CRP and improved inflammatory status, while others have not shown clear clinical benefit. These differences may reflect differences in baseline nutritional status (e.g., zinc in flaxseed), strain selection (e.g., Lactobacillus species), and pre-intervention gut microbiota structure. FMT is still exploratory, supported by animal studies and individual RA case reports. Currently, there is insufficient evidence to support the widespread application of FMT in the general RA population or specifically in EORA. However, this does not imply that FMT is ineffective in these populations, but rather highlights a critical research gap.

Due to the paucity of high-quality evidence specifically targeting EORA population, some conclusions in this review remain cautiously extrapolated from studies in general RA populations. These limitations constrain the interpretability and generalizability of the existing evidence. First, the majority of RA trials and observational studies have included older adults but without prespecified age-stratified subgroup analyses, making it uncertain whether the findings are equally applicable to the EORA population. Second, elderly patients often present with polypharmacy and multimorbidity, factors that may substantially influence the effectiveness of dietary or nutraceutical interventions. At the same time, it may be affected by confounding factors such as smoking, which is one of the strong environmental pathogenic factors of RA, and there are currently no studies to provide EORA-specific smoking stratification data. The influence of this confounding factor not only biased the findings, but also limited the comparability of the results across studies. Third, standard RA outcome measures, such as DAS28, HAQ, which were developed and validated in general RA populations. Their performance in elderly patients remains inadequately characterized. Therefore, other components of comprehensive assessment, including physical function limitations, cognitive status, and frailty, should be integrated into the routine management of EORA to enable comprehensive, patient-centered care. These measures capture the multidimensional health status of older adults more sensitively and address the information gap left by conventional RA core outcome measures in the EORA population.

## 6 Conclusion

The current management of RA across all age groups relies on antirheumatic drugs (csDMARDs, bDMARDs, tsDMARDs) to suppress inflammation and halt structural joint damage. However, the use of these conventional therapies in the EORA cohort is often limited by perceived or real risks of adverse events (e.g., infections, malignancies), cost, and, consequently, poor patient adherence or refusal, driven by concerns related to age and comorbidities (46, 145). This treatment gap creates an imperative to explore alternative or adjunctive strategies, such as microbiota-based therapies, which may offer a more tolerable and acceptable option for elderly patients. As reviewed here, a range of strategies targeting the gut microbiota have shown potential in modulating inflammatory pathways and improving disease activity (146). The field urgently needs comprehensive multi-omics profiling (gut metagenomics, immunosenescence markers, inflammaging biomarkers), specific RCTs or prospective studies of dietary and nutraceutical interventions in strictly defined EORA populations to validate these findings. A deeper understanding of the interplay between specific dietary components, the pre-existing gut microbial architecture, and host immune function will be crucial for developing personalized, microbiota-based adjunctive therapies that can safely and effectively bridge the current treatment gap in EORA.
